# The correlation between falls efficacy and activities of daily living among older adults receiving different types of care: a 2018–2019 cross-sectional study in Shanghai, China

**DOI:** 10.1186/s12889-023-15605-y

**Published:** 2023-04-23

**Authors:** Jian Wang, Qi Zhao, Zhipeng Li, Ting Yi Jen

**Affiliations:** 1 International Medical Center, Zhongshan Hospital, Fudan University, 130 Yixueyuan Road, Shanghai, 200032 China; 2 Department of General Practice, Zhongshan Hospital, Fudan University, Shanghai, 200032 China; 3 Department of Social Medicine, School of Public Health, Fudan University, Shanghai, 200336 China; 4 Department of Tuberculosis Control, Shanghai Municipal Center for Disease Control and Prevention, 1380 Hongqiao Road, Shanghai, 200336 China

**Keywords:** Falls efficacy, Activities of daily living, Stay at home, Senior day care center, Older adults

## Abstract

**Background:**

Falls in older adults has become a significant public health concern worldwide. Falls-related self-efficacy is closely related to healthy aging. This study investigated older adults receiving different types of care to clarify the correlation between falls efficacy and Activies of Daily Living (ADL), providing a theoretical basis for achieving healthy aging.

**Methods:**

An investigation comparing older adults attending senior day care centers and healthy older adults staying at home in the community was carried out by using structured questionnaires, including individual participants’ data, Falls Efficacy Scale International (FES-I), Patient Health Questionnaire-9, Generalized Anxiety Disorder Scale-7 and Lawton Instrumental Activities of Daily Living Scale (Lawton IADLs).

**Results:**

A total of 336 older adults were enrolled, and 153 (45.5%) older adults attending senior day care centers daily. The FES-I score of all the respondents was 30.65 ± 13.892, while the scores of healthy older adults staying at home in the community and attending senior day care centers were 25.05 ± 10.036 and 37.35 ± 14.894, respectively (p < 0.05). Among healthy older adults staying at home in the community, those using walking aids (OR = 53.595, 95%CI: 8.181, 351.129), with fear of falling (OR = 5.909, 95%CI:1.374, 25.407) and with anxiety symptoms (OR = 23.620, 95%CI: 6.077, 91.802) had low falls efficacy. Among older adults attending senior day care centers daily, those with higher education levels had high falls efficacy (OR = 0.276, 95%CI: 0.088, 0.862), and those with poor sleep quality (OR = 4.469, 95%CI: 0.682, 29.312), comorbidities (OR = 9.820, 95%CI: 1.990, 48.456), and with severe depressive symptoms (OR = 3.680, 95%CI: 1.098, 12.335) had low falls efficacy. The older adults with a higher score of Lawton IADLs had higher falls efficacy.

**Conclusions:**

Older adults attending senior day care centers daily had lower falls efficacy and needed to be paid more attention to in fall prevention. Targeted health promotion activities were necessary for older adults to improve their falls efficacy to achieve healthy aging.

**Strengths and limitations of this study**.

- There are limited studies regarding the correlation between falls efficacy and activities of daily living.
- There is no report on the correlation between falls efficacy and activities of daily living among older adults receiving different types of care.
- This cross-sectional study investigated older adults receiving different types of care from the perspective of falls efficacy and activities of daily living.
- Our study may provide a baseline to health care providers as a reference to improve falls efficacy in older adults and enhance their activities of daily living.
- This study was based on a cross-sectional design. Therefore, selection bias may exist, and the causal inference is unknown.

## Background

Population aging is one of the global social challenges in the 21st century. According to the Seventh National Population Census in 2020, China has 264 million people aged 60 and above, accounting for 18.7% of the population. Human wellbeing is crucial in coping with the aging society. The World Health Organization (WHO) regards healthy aging as an effectual development strategy for coping with the aging population. Viewing health from a life course perspective has prompted health workers to address and intervene risk factors that can affect the health, longevity, and quality of life in older ages, minimize risk factors, and enhance protective factors. In 1997, the G7 Summit in Denver proposed “active aging”; in 2002, the WHO created a policy framework,“Active Aging”. Active aging refers to maximizing opportunities for health, participation, and security to improve the quality of life as people age. It has gradually become a new theory, policy, and development strategy to deal with population aging in the 21st century.

The prevalence and incidence of diseases increase in older adults due to the decline of physiological functions and immunity [1–3]. Fall is the most common and significant injury faced by older adults worldwide. The high incidence and serious consequences of falls in older adults have become the leading public health concern [4]. Falls can lead to disability and functional incapacity and immensely affect the quality of life of older adults. Patients with a fall history had varying degrees of anxiety and depression. Anxiety and depression can affect the intrinsic function of older adults, leading to falls and limitations of the activities of daily living (ADL), which hinder the process of healthy aging.

The social cognitive theory argues that although physical functions show an irreversible decline, older adults have the advantages in knowledge, skills, and experience that can compensate for this decline to a certain extent. The key lies in evaluating their self-efficacy to facilitate the self-efficacy coping mechanism to take into effect. Many studies have shown that self-efficacy is closely related to active aging, which can promote positive coping skills or strategies in older adults, cultivate positive lifestyle changes, and shape healthy behaviors [5, 6].

In 1990, American scholar TINETTI et al. first proposed the concept of falls efficacy [7], which referred to the degree of confidence and self-perception that an individual would not fall during ADL. Older people with low falls efficacy had low confidence in not falling during activities and a high degree of fear of falling (FOF). A study by Li et al. [8] showed that the FOF affected the level of falls efficacy, and the reduction of falls efficacy further affected an individual’s functional status, which led to the limitation in older adults’ daily life, eventually affecting the quality of life profoundly. Substantial evidence shows that reducing or eliminating risk factors such as physical and emotional impairments can prevent falls [9].

However, there are very few domestic studies on falls efficacy and fewer studies on the correlation between falls efficacy and ADL. There is no report on the correlation between falls efficacy and ADL among older adults receiving different types of care in China. Hence, we conduct this study to investigate older adults receiving different types of care from the perspective of falls efficacy and ADL, to discover the influencing factors of falls efficacy and ADL, to clarify the correlation between falls efficacy and ADL, and as an attempt to improve their falls efficacy to improve their ADL, consequently achieve healthy aging.

## Methods

### Participants

In this study, we investigated two different groups of older adults. The senior day care group with 157 registered older adults was recruited from the Fushoukang Elderly Care Institution, which provided day care for people over 60 in the affiliated community between 2018 and 2019. The healthy stay-at-home group was recruited from the Fenglin community health service center in Xuhui District between 2018 and 2019 by convenience sampling. Finally, 153 older adults attending senior day care centers daily and 183 healthy older adults staying at home in the community were enrolled as participants.

Inclusion criteria: ① age ≥ 65 years; ② sufficient understanding of the Chinese language without communication barriers; ③ without long-term bedridden status; ④ willingness to participate with informed consent. Exclusion criteria: ① severe mental disorders; ② unable to cooperate with the investigation.

### Sample size

According to the formula.

$$n = {\left( {\frac{{{\mu _a}\sigma }}{\delta }} \right)^2}$$

N stands for the required sample size. µα stands for the µ value when the cumulative probability from left to right is 1-α (both sides) in the standard normal distribution.δstands for the allowable error. σ stands for the standard deviation.

Based on the presurvey results of 50 respondents, the FES-I score of the respondents was 30.10 ± 14.052 points,with significance set to α of 0.05,σ = 15,δ = 2, N = 219. Therefore, the total number of respondents is 219.

## Methods

The data were collected through face-to-face surveys by trained general practitioners. The survey questionnaire consisted of five parts:

General information: name, gender, age, education level, height, weight, chronic diseases, foot problems, fall history, walking aid use, and sleep quality.

Falls Efficacy Scale International (FES-I): FES-I contains 16 items, including the dimensions of indoor activities (cleaning the room, putting on and taking off clothes, preparing simple meals, bathing, getting up from a chair or sitting down on a chair, going up-and downstairs, answering a phone, reaching for objects high above head) and dimensions of outdoor activity (walking in the neighborhood, walking in the crowd, walking on slippery surfaces, walking on uneven surfaces, going up and down the slope, shopping, visiting relatives and friends, participating in social activities). Respondents circled the option best reflected their concern about the likelihood of falling when engaging in each activity. Responses for each item were measured on a 4-point Likert scale: (1) not at all concerned, (2) somewhat concerned, (3) fairly concerned, and (4) very concerned. The total score ranges from 16 to 64 [10]. The higher the total score, the more concerned he/she was about falling, the greater his/her FOF, the lower his/her falls efficacy [7]. The Cronbach’s alpha of FES-I was 0.94 [10].

Patient Health Questionnaire-9: It contains 9 items about how the respondent felt in the past two weeks, and each item was measured on a 4-point Likert scale: (1) no depressive symptoms, (2) depressive symptoms occur several days, (3) depressive symptoms occur more than seven days, (4) depressive symptoms occur every day. The total score ranges from 0 to 27. Scores of 0 to 4 indicate no depression symptoms, scores of 5 to 9 indicate mild depression symptoms, scores of 10 to 14 indicate moderate depression symptoms, scores of 15 to 19 indicate moderate to severe depression symptoms, and scores of 20 to 27 indicate severe depression symptoms [11].

Generalized Anxiety Disorder Scale-7: It contains 7 items of how the respondent felt in the past two weeks, and each item was measured on a 4-point Likert scale: (1) no anxiety symptoms, (2) anxiety symptoms occur several days, (3) anxiety symptoms occur more than seven days, (4) anxiety symptoms occur every day. The total score ranges from 0 to 21. Scores of 0 to 4 indicate no anxiety symptoms, scores of 5 to 9 indicate mild anxiety symptoms, scores of 10 to 14 indicate moderate anxiety symptoms and scores of 15 to 21 indicate severe anxiety symptoms [11].

Lawton Instrumental Activities of Daily Living Scale (Lawton IADLs): Lawton IADLs include two sub-scales, the Physical Self-Maintenance Scale (PSMS) and the Instrumental Activities of Daily Living Scale (IADL). The PSMS includes 6 items: toilet, feeding, dressing, grooming, physical ambulation, and bathing. Each item has 5 options, and each option scored 0–1 point. The score of PSMS ranges from 0 to 6 points. A higher score is associated with better ADL. The IADL includes 8 items: the ability to use the telephone, shopping, food preparation, housekeeping, laundry, mode of transportation, responsibility for own medications, and ability to manage personal finances. Each item has 3 to 5 options, and each option is scored 0 to 1 point. In some items, only the highest level of functioning scored 1 point. In other cases, two or more levels scored 1 point. The score of IADL ranges from 0 (low functioning, dependent) to 8 (high functioning, independent). The total score of Lawton IADLs ranges from 0 to 14. The Cronbach’s alpha of Lawton IADLs was 0.96 [12].

### Data analysis

We used Excel to establish the database and SPSS 20.0 for data processing and statistical analysis. Quantitative data were expressed as mean ± standard deviation (x ± s), and variance analysis or t-test was used for comparison; qualitative data were expressed by the constituent ratio (%), and a chi-square test was used for comparison. Multivariant analysis was conducted by binary logistic regression, and P < 0.05 was considered statistically significant. Multiple linear regression models were employed in the multivariate analysis to identify risk factors for falls efficacy. Gender, age, marriage, education level, use of walking aid, sleep quality, comorbidities, foot problems, fear of falling, depressive symptoms, and anxiety symptoms were included as independent variables in linear regression models as covariates. Binary logistic regression models were employed in the multivariate analysis to study the effect of ADL on falls efficacy. Gender, age, marriage, education level, use of walking aid, sleep quality, comorbidities, foot problems, fear of falling, depressive symptoms, and anxiety symptoms were included in the multivariable models as covariates.

## Results

### Baseline characteristics of participants

This study included 183 healthy older adults staying at home in the community and 153 older adults attending senior day care centers daily. One or two comorbidities were the most common, accounting for 69.9% and 56.9%, respectively. Most participants did not have foot problems, accounting for 85.8% and 68.6%, respectively. The majority of the participants did not use walking aids, accounting for 88.5% and 57.5%, respectively. Most participants had secondary education and above, accounting for 83.6% and 51.0%, respectively. The majority of the healthy older adults staying at home were 65–74 years old (53.0%) and married (81.4%). The majority of older adults attending senior day care centers daily were 85 years old or above (67.3%), single, divorced, or widowed (74.5%) (Table 1).

**Table 1:** Baseline characteristics of participants

Variable		Staying at home N (%)	Attending senior day care centers N (%)	Chi-square	P-value
Gender	Female	117 (63.9)	107 (69.9)	1.350	0.245
	Male	66 (36.1)	46 (30.1)		
Age	65–74	97 (53.0)	12 (7.8)	113.488	< 0.001
	75–84	59 (32.2)	38 (24.8)		
	85 and above	27 (14.8)	103 (67.3)		
Marital status	Single,divorced,widowed	34 (18.6)	114 (74.5)	105.770	< 0.001
	Married	149 (81.4)	39 (25.5)		
Education level	Primary education and below	30 (16.4)	75 (49.0)	41.287	< 0.001
	Secondary education and above	153 (83.6)	78 (51.0)		
Walking aid	Not used	162 (88.5)	88 (57.5)	42.072	< 0.001
	Used	21 (11.5)	65 (42.5)		
Sleep quality	Good	90 (49.2)	66 (43.1)	1.418	0.492
	Fair	69 (37.7)	67 (43.8)		
	Poor	24 (13.1)	20 (13.1)		
Comorbidity	None	18 (9.8)	31 (20.3)	8.714	0.013
	1 to 2	128 (69.9)	87 (56.9)		
	3 or more	37 (20.2)	35 (22.9)		
Foot problem	No	157 (85.8)	105 (68.6)	14.297	< 0.001
	Yes	26 (14.2)	48 (31.4)		
Depressive symptoms	None or mild	61 (33.3)	44 (28.8)	0.812	0.368
	Moderate and above	122 (66.7)	109 (71.2)		
Anxiety symptoms	None or mild	143 (78.1)	82 (53.6)	22.698	< 0.001
	Moderate and above	40 (21.9)	71 (46.4)		

*Foot problem:Corns with pain; Diabetic foot; Gout; Flat feet; Swelling of ankle or foot

### Falls efficacy scores of the older adults receiving different types of care

The FES-I score of the respondents was 30.65 ± 13.892 points, among which the scores of the healthy older adults staying at home and attending senior day care centers daily were 25.05 ± 10.036 and 37.35 ± 14.894 points, respectively, and the difference was statistically significant.

Respondents with FES-I scores ≥ 28 points were highly concerned about falling, and their falls efficacy was extremely low [13]. Among all the respondents, 157 older adults had FES-I ≥ 28, accounting for 46.7% (157/336); 49 stay-at-home healthy older adults had FES-I ≥ 28 points, accounting for 26.8% (49/183); 108 older adults attending senior day care centers daily had FES-I ≥ 28 points, accounting for 70.6% (108/153).

Regardless of locations of care, the FES-I scores of older adults using walking aids were significantly higher than those who did not (t=-5.991, P < 0.001; t=-6.782,P < 0.001). The FES-I scores of those with FOF were significantly higher than those without FOF (t=-6.019,P < 0.001;t=-3.056,P = 0.003). The FES-I scores of older adults with moderate or severe depressive symptoms or anxiety symptoms were significantly higher than those with no or mild depressive symptoms (t=-5.830,P < 0.001;t=-6.186,P < 0.001) or anxiety symptoms (t=-5.002,P < 0.001;t=-5.297,P < 0.001).

Among healthy older adults staying at home in the community, the older their age, the higher their FES-I scores and the more concerned they were about falling (F = 17.496,P < 0.001). Single, divorced, or widowed healthy older adults had higher FES-I scores and were more concerned about falling than those who were married (t = 2.304,P = 0.027). For older adults attending senior day care centers daily, those with poor sleep quality and multiple comorbidities had higher FES-I scores (Table 2).

**Table 2:** Falls efficacy scores of older adults receiving different types of care

Variable		Staying at home				Attending senior day care centers		
		Score	t (F) value	P value		Score	t (F) value	P value
Gender	Female	24.36 ± 9.659	-1.240	0.216		36.64 ± 15.122	-0.888	0.376
	Male	26.27 ± 10.637				38.98 ± 14.378		
Age	65–74	22.57 ± 8.083	17.496	< 0.001		33.25 ± 12.447	0.797	0.453
	75–84	24.83 ± 7.403				36.08 ± 14.372		
	85 and above	34.44 ± 15.062				38.29 ± 15.344		
Marriage	Single,divorced,widowed	29.76 ± 14.071	2.304	0.027		36.98 ± 14.522	-0.516	0.607
	Married	23.97 ± 8.570				38.41 ± 16.082		
Education level	Primary education and below	27.90 ± 12.254	1.710	0.089		39.13 ± 14.297	1.461	0.146
	Secondary education and above	24.49 ± 9.489				35.63 ± 15.340		
Walking aid	Not used	22.93 ± 7.117	-5.991	< 0.001		31.18 ± 12.998	-6.782	< 0.001
	Used	41.38 ± 13.876				45.69 ± 13.197		
Sleep quality	Good	24.37 ± 9.389	0.541	0.583		29.82 ± 13.664	19.068	< 0.001
	Fair	25.39 ± 10.462				42.12 ± 13.124		
	Poor	26.63 ± 11.298				46.20 ± 13.336		
Comorbidity	None	28.00 ± 14.701	2.172	0.117		25.90 ± 9.894	17.252	< 0.001
	1 to 2	24.05 ± 9.488				38.25 ± 14.552		
	3 or more	27.05 ± 8.810				45.23 ± 13.571		
Foot problem	No	25.29 ± 10.645	1.307	0.195		36.09 ± 13.573	-1.423	0.159
	Yes	23.62 ± 4.875				40.10 ± 17.275		
FOF	No	20.31 ± 4.453	-6.019	< 0.001		35.18 ± 14.757	-3.056	0.003
	Yes	27.25 ± 11.106				43.27 ± 13.766		
Depressive symptoms	None or mild	20.52 ± 4.414	-5.830	< 0.001		26.84 ± 11.897	-6.186	< 0.001
	Moderate and above	27.31 ± 11.241				41.59 ± 13.883		
Anxiety symptoms	None or mild	22.55 ± 6.833	-5.002	< 0.001		31.88 ± 13.570	-5.297	< 0.001
	Moderate and above	33.98 ± 13.983				43.66 ± 13.896		

Multiple linear regression analysis found that the characteristics related to the falls efficacy differed among the older adults receiving different types of care. Among the healthy older adults staying at home in the community, those using walking aids (OR = 53.595, 95%CI:8.181, 351.129), with FOF (OR = 5.909, 95%CI:1.374, 25.407), and with anxiety symptoms (OR = 23.620, 95%CI:6.077, 91.802) had high falls efficacy scores and low falls-related self-efficacy. Falls efficacy among older adults persons attending senior day care centers daily was related to education level, sleep quality, types of comorbidities, and the degree of depressive symptoms. Older adults with high education levels had low falls efficacy scores and high falls-related self-efficacy (OR = 0.276, 95%CI:0.088, 0.862). Those with poor sleep quality (OR = 4.469, 95%CI:0.682, 29.312), multiple types of comorbidities (OR = 9.820, 95%CI:1.990, 48.456), and severe depressive symptoms (OR = 3.680, 95%CI:1.098, 12.335) had high falls efficacy scores and low falls-related self-efficacy (Table 3).

**Table 3:** Characteristics affecting falls efficacy among the older adults **receiving different types of care**

Variable		Staying at home				Attending senior day care centers		
		OR	95% CI			OR	95% CI	
Gender	Male: female	2.095	0.615	7.132		2.198	0.637	7.584
Age	75 to 84: 65 to 74	1.271	0.406	3.985		0.684	0.067	6.936
	85 and above: 65 to 74	3.003	0.627	14.384		0.963	0.104	8.934
Marital status	Married: single, divorced,widowed	0.522	0.135	2.023		1.288	0.374	4.443
Education Level	Secondary education and above: primary education and below	0.549	0.131	2.302		0.276	0.088	0.862
Walking aid	Used: not used	53.595	8.181	351.129		2.612	0.814	8.382
Sleep quality	Fair: good	1.548	0.488	4.910		6.744	2.168	20.981
	Poor: good	0.505	0.095	2.684		4.469	0.682	29.312
Comorbidity	1 to 2: none	0.729	0.087	6.142		7.204	2.022	25.668
	3 or more: none	5.291	0.526	53.178		9.820	1.990	48.456
Foot problem	Yes: no	0.364	0.068	1.933		0.214	0.059	0.774
FOF	Yes: no	5.909	1.374	25.407		1.800	0.377	8.590
Depressive symptoms	Moderate or severe: none or mild	1.780	0.467	6.791		3.680	1.098	12.335
Anxiety symptoms	Moderate or severe: none or mild	23.620	6.077	91.802		1.885	0.577	6.157

### ADL of the older adults

Regarding ADL in all the participated older adults, the Lawton IADLs scores were 10.53 ± 4.452 points, the PSMS scores were 4.45 ± 2.146 points, and the IADLs scores were 6.07 ± 2.532 points. The scores for the healthy older adults staying at home in the community and attending senior day care centers daily were 12.48 ± 3.012 and 8.20 ± 4.773 points, respectively, and the difference was statistically significant.

Binary logistic regression was used to study the effect of ADL on falls efficacy by taking an FES-I score ≥ 28 points as the dependent variable. The results showed that regardless of the adjusted influential characteristics like age and gender, older adults with a higher score of Lawton IADLs had higher falls efficacy and less FOF. After adjusting for age, gender, and other influential characteristics, for each increased point in Lawton IADLs score, the possibility of high falls efficacy increased by 2.020 times for the healthy older adults staying at home in the community and 1.706 times for the older adults attending senior day care centers, respectively (Table 4).

**Table 4:** Multivariable association between ADL and falls efficacy among the older adults receiving different types of care

	Variable		Staying at home				Attending senior day care centers		
			OR	95% CI			OR	95% CI	
MODEL 1	The Lawton IADLs scores		0.470	0.356	0.622		0.605	0.507	0.722
MODEL 2	The Lawton IADLs scores		0.500	0.378	0.660		0.590	0.488	0.713
	Gender	Male: female	1.226	0.500	3.007		0.795	0.271	2.331
	Age	75 to 84: 65 to 74	1.503	0.575	3.928		1.781	0.256	12.375
		85 and above: 65 to 74	2.205	0.609	7.986		2.643	0.413	16.899
MODEL 3	The Lawton IADLs scores		0.495	0.318	0.772		0.586	0.456	0.754
	Gender	Male: female	1.242	0.297	5.195		0.652	0.140	3.026
	Age	75 to 84: 65 to 74	1.270	0.351	4.599		0.748	0.027	20.608
		85 and above: 65 to 74	1.168	0.161	8.489		1.050	0.038	28.869
	Marriage	Married: single, divorced,widowed	0.432	0.095	1.953		0.854	0.190	3.842
	Education level	Secondary education and above: primary education and below	0.714	0.148	3.440		0.373	0.092	1.512
	Walking aid	Used: not used	8.196	0.731	91.824		0.759	0.198	2.907
	Sleep quality	Fair: good	1.286	0.334	4.950		3.926	1.036	14.873
		Poor: good	0.351	0.052	2.378		1.641	0.189	14.244
	Comorbidity	1 to 2: none	0.225	0.027	1.903		6.460	1.449	28.795
		3 or more: none	1.748	0.183	16.733		7.308	1.038	51.479
	Foot problems	Yes: no	0.566	0.097	3.315		0.086	0.014	0.518
	FOF	Yes: no	7.906	1.592	39.273		2.609	0.421	16.182
	Depressive symptoms	Moderate and severe: none or mild	0.998	0.236	4.218		4.040	0.961	16.985
	Anxiety symptoms	Moderate and severe: none or mild	31.272	6.653	146.989		0.484	0.095	2.469

* Model 1: unadjusted;

Model 2: Adjusted by gender and age;

Model 3: Adjusted by gender, age, marriage, education level, use of walking aid, sleep quality, comorbidities, foot problems, fear of falling, depressive symptoms, and anxiety symptoms

## Discussion

Our study found that older population had lower falls efficacy, especially the older adults attending day care centers daily. Walking aids, fear of falling, anxiety symptoms, poor sleep quality, comorbidities and severe depressive symptoms increase the fall risk. According to the recommondations of the FES-I evaluation, the scores of 20–27 and 28–64 were defined as moderate concern and high concern about falling, respectively [13]. In this study, the average FES-I scores of the respondents were 30.65 ± 13.892, indicating highly concerned about falling when they were engaged in the 16 items of daily activities. We found that the score of the older adults attending senior day care centers daily was significantly higher than the healthy old adults staying at home in the community (37.35 ± 14.894 vs. 25.05 ± 10.036), which indicated that they were more afraid of falling and had lower falls-related self-efficacy [7]. Kuo et al. evaluated 751 community-based older adults in Taipei, which showed that the average FES-I scores were 23.5 ± 8.99 [14]. Different characteristics of our respondents led to a higher average FES-I score compared to Kuo’s results. Older adults receiving different types of care lead to different concerns about falling. Our study suggested that we should also pay greater attention to older adults attending senior day care centers daily as they had higher FES-I scores. It is necessary to reinforce health education on fall prevention and conduct psychological counseling to overcome FOF and improve falls efficacy of older adults.

The older adults who used walking aids tended to have a more significant decline in physical function than those without walking aids, hence they were more concerned about falling. Exercise intervention is necessary for walking aids users to increase their muscle strength and balance, overcome the fear of falling, and consequently improve their falls efficacy. Hull et al. reported that use of walking aids was the most significant predictor variable for models associated with FES-I in the older population [15]. Another study of 1088 older people by Kumar et al. illustrated that walking aid was a risk factor for high FOF and low falls-related self-efficacy [16]. Consistent with previous studies, our study showed similar results: using walking aids had higher FES-I scores and lower falls-related self-efficacy.

FOF is prevalent among community-dwelling older adults domestically and globally [17–19]. People with low falls efficacy have high FOF and low confidence in their ability to perform activities of daily living without falling [20]. FOF can be part of a vicious fall-associated circle as it can lead to activity restriction, further decline in physical functioning, greater fall risk, and admission to institutional care [7, 9, 10]. A study on 420 older adults in community found that FOF was correlated with falls efficacy [21]. In KEMPEN et al.‘s study in Germany, Netherlands, and UK, he concluded that FOF was strongly associated with FES-I scores [22]. In Hellstrom’s study, patients with FOF had lower falls efficacy (P = 0.001) [23]. These domestic and foreign research results were consistent with our results. We should focus on the FOF among home-dwelling older adults, provide adequate psychological support, reduce or eliminate their FOF, and increase their falls efficacy.

Previouse research found that anxiety was independently associated with falls efficacy [15, 24]. Payette et al.’s study was the first meta-analysis on the relationship between anxiety and fall-related psychological concerns among community-dwelling older adults [25]. Random-effect meta-analysis revealed that the mean effect size for falls efficacy and anxiety was r = 0.31 (95%CI: 0.23, 0.40), Z = 6.72, P < 0.001 [25]. Our study also demonstrated that anxiety was related to low falls efficacy.

We found that older adults attending senior day care centers daily with high education levels had high falls efficacy (OR = 0.276, 95% CI:0.088, 0.862),which was the same as other studies. In 2014, Zheng et al. reported that Modified Fall Efficacy Scale scores were positively associated with education (r = 0.234,P = 0.011). Participants with higher education levels had more confidence in their ability to avoid falling [26]. Shin et al. found that more educated older adults reported lower scores on the FOF[27]. The low falls efficacy of older adults with low education levels may be related to a lack of knowledge. Therefore, an easy-to-understand and practical education program should be considered to increase their knowledge, reduce or remove their FOF, and improve their falls efficacy.

Better sleep results in more energy, physical strength, better balance control, and less FOF. We found that older adults attending senior day care centers with poor sleep quality had low falls efficacy, which was consistent with the study on older adults in nursing homes in Chengdu [28], the survey on elderly patients after orthopedic surgery [29], and the study on community-dwelling older adults in South Korea [27].

Many studies showed that depressive symptoms were related to falls efficacy [30, 31]. It was found that severe depressive symptoms had low falls efficacy. Older adults with greater FOF are inclined to report depressive symptoms more frequently. Our study also found that severe depressive symptoms increase the risk of fall. Depression may disturb one’s sense of independence and confidence in performing activities, making older adults concentrate on their physical abilities and resulting in FOF.

The Lawton IADLs score of our respondents was 10.53 ± 4.452 points (total score 0 to 14 points), indicating that the ability of their ADL had decreased. In China, older adults incapable of performing ADL independently would like to attend senior day care centers. Our study showed that the Lawton IADLs score of the older adults attending senior day care centers daily (8.20 ± 4.773 points) was lower than the older adults staying at home (12.48 ± 3.012 points), which was consistent with the aging process. Our multiple regression analysis indicated that the higher the Lawton IADLs score, the higher the falls efficacy and the less FOF. Yan et al. studied older adults in nursing homes and found that ADL was correlated with falls efficacy (r = 0.413,P < 0.001) [28]. Guo et al. demonstrated that ADL was associated with falls efficacy among older adults in the community (P = 0.035) [21]. Shin et al. studied 213 South Korean community-dwelling older adults and found that their FOF was affected by ADL [27]. Grönstedt et al. analyzed 322 residents from nursing homes in Sweden, Norway, and Denmark to conclude that participants with a high dependency on ADL had lower fall-related self-efficacy than less ADL-dependent participants [32]. All these domestic and foreign studies amongst older adults indicated that lower physical functions would lead to low ADL and low falls efficacy. Targeted health promotion activities were necessary to enhance their ADL and improve their falls efficacy.

## Limitation

When interpreting the results of the study, the limitation of sample selection should be considered. The baseline characteristics of the study participants were not evenly distributed between the two groups, which increased the possibility of selection bias. As the study was based on a cross-sectional design, a causal relationship could not be inferred with certainty.

We conducted the survey at Fushoukang Elderly Care Institution, one of the largest institutions that provide day care for people over 60 years old in a community in Shanghai. The result of our study may only apply to specific areas in Shanghai. In the future, we can also compare and analyze older adults in areas with different economic and cultural backgrounds to verify the credibility of this study result.

## Conclusions

Older adults attending senior day care centers daily had lower falls efficacy and needed to be paid more attention to in fall prevention. Targeted health promotion activities were necessary for older adults to improve their falls efficacy to achieve healthy aging.

## Data Availability

The datasets generated and/or analysed during the current study are not publicly available due to the university policy as this manuscript is part of my PhD thesis that the datasets are confidential but are available from the corresponding author on reasonable request.

## Abbreviations

WHO: World Health Organization
ADL: Activities of Daily Living
FOF: Fear of Falling
FES-I: Falls Efficacy Scale International
Lawton IADLs: Lawton Instrumental Activities of Daily Living Scale
PSMS: Physical Self-maintenance Scale
IADL: Instrumental Activities of Daily Living Scale
